# Studying additive interaction in a healthcare database: Case study of NSAIDs, cardiovascular profiles, and acute myocardial infarction

**DOI:** 10.1371/journal.pone.0201884

**Published:** 2018-08-10

**Authors:** Michèle Bally, Lyne Nadeau, James M. Brophy

**Affiliations:** 1 Department of Pharmacy and Research Center, University of Montreal Hospital, Montreal, Canada; 2 Centre for Outcomes Research and Evaluation, Research Institute of the McGill University Health Centre, Montreal, Canada; 3 Department of Epidemiology, Biostatistics, and Occupational Health, McGill University, Montreal, Canada; 4 Department of Medicine, McGill University Health Centre, Montreal, Canada; University of Manitoba, CANADA

## Abstract

**Purpose:**

There are clinical trial data on risk of acute myocardial infarction (MI) with nonsteroidal anti-inflammatory drugs (NSAIDs) in patients at increased cardiovascular (CV) risk requiring chronic daily treatment. This study investigated whether risks of acute MI with real-world prescription NSAIDs, such as low-dose or intermittent use, vary according to an individual’s CV profile.

**Methods:**

Nested case-control analyses were carried out on an administrative health cohort from Quebec, Canada by randomly selecting 10 controls per case matched on age ± 1 year, sex, and month and year of cohort entry. We measured the additive joint effects on acute MI of current NSAID use and presence of hypertension, coronary heart disease (CHD), history of previous MI, or concomitant use of cardioprotective aspirin. The endpoint was the relative excess risk due to interaction (RERI). To verify the robustness of interaction findings, we performed sensitivity analyses with varying specifications of NSAID exposure-related variables.

**Results:**

The cohort consisted of 233 816 elderly individuals, including 21 256 acute MI cases. For hypertension, CHD, and previous MI, we identified additive interactions on MI risk with some but not all NSAIDs, which also depended on the definition of NSAID exposure. Hypertension was sub-additive with naproxen but not with the other NSAIDs. Celecoxib and CHD were sub-additive in the primary analysis only (modelling NSAID dose on index date or up to 7 days before–best-fitting base model) whereas celecoxib and rofecoxib were super-additive with a history of previous MI in the secondary analysis only (modelling NSAID use on index date). For cardioprotective aspirin we found no evidence for an additive interaction with any of the NSAIDs.

**Conclusions:**

Alternative specifications of NSAID exposure concurred in finding that concomitant use of cardioprotective aspirin does not attenuate the risks of acute MI with NSAIDs. However we were unable to demonstrate consistent interactions between an individual’s cardiovascular comorbidities and NSAID-associated acute MI. Our study highlights challenges of studying additive interactions in a healthcare database and underscores the need for sensitivity analyses.

## Introduction

Clinical practice guidelines advise that patients with major risk factors or a diagnosis of cardiovascular (CV) disease are particularly vulnerable to the cardiotoxicity of nonsteroidal anti-inflammatory drugs (NSAIDs).[1, 2] The large Prospective Randomized Evaluation of Celecoxib Integrated Safety vs. Ibuprofen Or Naproxen (PRECISION) trial [3] determined the relative safety of celecoxib, compared with ibuprofen and naproxen in patients who were at increased cardiovascular risk. However it involved patients who required chronic daily treatment with NSAIDs, which may not represent the reality of many patients who have a CV comorbidity but only use NSAIDs in low doses or intermittently.[4, 5] Whether the risk of acute myocardial infarction (MI) with a specific NSAID used in real-world conditions is modulated by an underlying CV comorbidy is a question that clinicians would like answered.

We investigated the possible presence of additive interactions for MI between each common NSAID and (i) history of previous MI, (ii) coronary heart disease without a previous MI (CHD), (iii) hypertension, or (iv) concomitant use of cardioprotective aspirin. We based our study on administrative health data as they are well suited to characterize real-world drug safety.[6]

## Methods

The study was approved by the McGill University Health Centre Research Ethics Board (13-380-SDR). To inform public health and clinical decision-making, we studied interaction on the additive scale. [7–9] Since the findings of interaction studies are not immune to biases, [7] we undertook to minimize the possibility of confounding and performed sensitivity analyses with varying specifications of NSAID exposure-related variables.

### Study design and data source

We used the universal public insurance databases of Quebec, Canada (Régie de l'assurance maladie du Québec, RAMQ). Each person’s identifier allowed linking individual data involving demographic information, medical services claims, dispensed outpatient prescription drugs, hospitalization data, indicator of hospital mortality, and long term vital statistics. These databases were shown to be valid for this purpose [10, 11] including for cardiovascular research.[12, 13]

We performed an individually-matched nested case-control analysis by randomly selecting 10 controls matched on age ± 1 year, sex, and month/year of cohort entry. Hospital admission date for acute MI was the index date for cases. For their controls, the date that resulted in the same cohort follow-up time was the assigned index date, thereby controlling for potential calendar time effects.

We have reported a nested case-control analysis of NSAID and MI risk, based on the same dataset. That study [14] aimed to characterize the temporal association between time‐varying NSAID exposure and acute MI and provided evidence for the dose relationship, precise timing of risk onset, and the existence of a cumulative effect. This nested case-control dataset was also included in our previously published patient-level meta-analysis of population-based studies [15] therefore these works share some methodological aspects.

### Study participants

We assembled a cohort of elderly (≥66 years) new NSAID users (first time users or newly treated after a one-year baseline). To be included in this study, patients were required to have a prescription fill for a single NSAID after a one-year baseline and at least one year of history in RAMQ databases. Date of cohort entry was the first NSAID prescription after study start (1 January 1993) and cohort exit was the earliest of the following dates: study outcome, death, end of insurance coverage, or study end (30 September 2004). We choose this calendar time frame to minimize confounding by indication or by contraindication and allow for comparison with rofecoxib, which is important given the consistency and strength of randomized controlled trial evidence for MI risk with this drug.[16–19]

### Outcome ascertainment

The outcome was the first hospitalization for acute MI, ICD-9[20] code 410.x (positive predictive value 0.979 (95%CI: 0.970, 0.985).[12] For non-fatal MI cases, we used a validated definition [21, 22] corresponding to local practice relevant to study years. Length of hospital stay had to be at least 3 days, unless the patient was transferred to or from another institution or underwent percutaneous coronary angioplasty.

### Drug exposure ascertainment

NSAID use was determined as a time-varying exposure for each day of follow-up for the following common drugs: celecoxib, diclofenac, ibuprofen, naproxen, rofecoxib. All other prescription non-aspirin NSAIDs,(diflunisal, etodolac, fenoprofen, flurbiprofen, ketoprofen, indomethacin, mefenamic acid, meloxicam, nabumetone, piroxicam, sulindac, tenoxicam, tiaprofenic acid, and tolmetin sodium) entered nested case-control MI risk models as a group. The low prevalence of prescriptions for ‘other NSAIDs’ precluded studying interactions individually for these drugs. Use of over-the-counter (OTC) ibuprofen or aspirin is not captured in the RAMQ database.

Computer-recorded variables allowed the direct calculation of estimated daily dose of the common NSAIDs as pill strength times number of pills divided by number of days supplied. Days supplied and consecutive prescription dates confirmed the duration of each dispensing and allowed identifying gaps between the end of a prescription and the start of a next one–thereby allowing to characterize episodes of continuous use and non-use. A priori rules (Tables A and B in S1 File) were specified to capture behaviors such as intermittent use, dose changes, and drug switches such that patients could not be concurrently exposed to more than one NSAID over the study period. Ascertainment of prescription aspirin, which is captured in RAMQ, was done similarly to NSAID exposure. Medication adherence is a strong determinant of effective cardioprotection[23] such that we allowed a grace period of 7 days between two refills when defining continuous aspirin exposure status. Indication for cardioprotection was assumed for dosages ranging from 80 mg every other day to 650 mg daily.[24]

### Assessment and measurement of covariates

We [14, 15]identified risk factors for the outcome and potential confounders based on substantive knowledge and literature search.[25–28] Using a simplified causal graph[29] we mapped relationships between variables,[30] including time-dependent confounders that are mediating intermediates on the causal pathway between NSAID exposures and acute MI,[31] then confirmed the final set of confounders (Figure A in S1 File). Comorbidities were defined according to validation studies [32–37] and treatment guidelines.[38–41] We relied on ICD-9 codes recorded during hospitalization and on outpatient medications reflecting diagnoses recorded in the course of ambulatory care (Table C in S1 File). To increase specificity for hypertension, coronary heart disease, congestive heart failure, or rheumatoid arthritis, we used algorithms based on drug dispensing dates. The presence of a comorbidity was confirmed using the first occurrence of ICD-9 codes in the hospital discharge summary or by dispensed drugs over the cohort period preceding the index date, with the following exceptions: (i) potentially mediating comorbidities–hypertension, congestive heart failure, and renal failure–were assessed only before cohort entry, and (ii) ambulatory claims for comorbidities without any algorithm to overcome the low specificity of drug treatment–chronic pulmonary obstructive disease and gastrointestinal ulcer disease–were considered for the one year preceding the index date. Treatment with oral corticosteroids, clopidogrel, and cardioprotective aspirin was ascertained for the 30-day period prior to index date (Figure B in S1 File).

### NSAID exposure and nested case-control analysis

We prespecified two alternative definitions of time windows for exposure to each common NSAID (celecoxib, diclofenac, ibuprofen, naproxen, rofecoxib) and to other NSAIDs grouped together. In the first model, the mutually exclusive binary indicators of use for each NSAID were: (i) current use on the index date, (ii) recent use 1–30 days ago, (iii) past use 31–180 days ago, or (iv) no use in last 180 days before the index date. In the second model, (i) current use corresponded to index date or up to 7 days before, (ii) recent use was 8–30 days ago, and (iii) and (iv) were identical to above. Categories were assessed from (i) to (iv) for each NSAID, and once a category was set to 1 the subsequent categories were set to 0, ensuring they were mutually exclusive. These two models were repeated by replacing the current use in (i) by current daily dose (continuous variable).

A nested case-control analysis of this large cohort was chosen because it allows to study the additivity of risks[42] and is convenient from a computational standpoint[43]. First, we estimated the odds ratio (OR) of acute MI for NSAID exposure-related variables described above by conditional logistic regression, for each NSAID versus non- use of the NSAID in last 180 days before the index date, while adjusting for exposure to the other NSAIDs and potential confounders. The fit of models was compared through the Akaike Information Criterion (AIC).[44]

### Measures of additive interaction

We considered the joint effect of NSAIDs and CV risk profiles in absolute terms, thereby investigating interactions on the additive scale.[7–9] This has the advantage of allowing to consider risk in the presence of two exposures in absolute rather than in relative terms thus answering the question of whether the number of acute MIs in current NSAID users with certain CV risk profiles is greater or less than MI numbers expected from the additive effects of each separate risk factor. A finding of super-additivity (i.e. greater than additive joint effects) means that the number of acute MI cases due to an NSAID in the presence of a CV comorbidity is greater than the sum number of MI cases arising independently from either the NSAID exposure or the CV comorbidity.[9] We pre-specified that the primary analysis of additive interaction between NSAIDs and CV risk profiles would be based on the NSAID exposure parametrization giving the best fit to data in a model without product terms (base model). To verify the robustness of interaction findings, in a secondary analysis, we repeated our investigation this time using the NSAID exposure parametrization that had worst fit to data in a base model. Additionally, we checked whether a lower prevalence of CHD due to definition by stricter clinical criteria affected the results of additive interaction between CHD and NSAIDs. After model building and selection of the best model with product interaction terms, we obtained results for the relative excess risk due to interaction (RERI) and its 95%CI from exponentiated regression coefficients, directly from Stata. [42] The null hypothesis for exact additivity (i.e. no additive interaction) is RERI = 0. An upper 95% confidence interval (CI) bound for RERI <0 indicates sub-additive joint effects whereas a lower 95% CI bound for RERI >0 indicates super-additive joint effects.[45] (see also footnote to table of findings) The statistical analysis allowed for evaluating all interaction pairs and therefore accounted for the fact that an individual might have more than one CV risk. However, three-way interactions were not studied.

### Testing for additive interactions

Assessment of interactions first required fitting nested case control models consisting of (i) the specification of current NSAID exposure-related variables (ii) the binary time-varying indicators for recent and past use of other NSAIDs and (iii) the values of all potential confounders, which included the CV risk profiles of interest (either time-invariant or updated values of time-varying confounders, see Figure B and Table C in S1 File), and (iv) the binary product terms for each NSAID and each CV risk profile of interest.

We analyzed three datasets, corresponding to (a) the best-fitting model without product terms (base model), (b) the worst-fitting base model, and (c) a sensitivity analysis of lower prevalence of CHD in the best-fitting base model. With a Wald test, we checked the statistical significance of product terms when added to the base model in (a), (b), and (c), first for each product term individually, then with all product terms included in the base model. For each product term, we obtained RERI (95%CI) and the 95%CI for the exponentiated coefficient (β_11_). We then proceeded with model building, keeping in the first round all product terms which, when tested individually or together i) had a p-value of 0.2 or less; or ii) deviated or approximately deviated from exact multiplicativity or exact additivity. Interactions occur on a continuum of additivity and multiplicativity of risks.[46] Therefore, our systematic approach ensured that an interaction on the additive scale was not ignored by dismissing it on the basis of finding exact multiplicativity. We obtained more parsimonious models by deleting product terms that showed exact additivity and exact multiplicativity of joint effects of the NSAID and the CV risk profile. For each dataset in (a), (b), and (c), we compared models with product terms and selected the best-fitting based on the AIC.

## Results

Analyses were carried out with 233 816 individuals, of which 21 256 were acute MI cases (Figure C in S1 File). Table 1 presenting confounders at index date indicates that this older cohort (mean age 77.8 ±6.1 years) had a high baseline coronary risk.

**Table 1:** Prevalence of confounders at index date in nested case-control analyses of acute MI with NSAIDs in a RAMQ cohort of elderly individuals.

Confounder	Cases (n = 21 256)	Controls (n = 212 560)
Demographic characteristics*		
Age at cohort entry, years, mean (SD)	77.8 (6.1)	77.8 (6.1)
Male sex, %	50.7	50.7
**Comorbidities**^†^		
Diabetes, %	28.5	16.3
Hyperlipidemia, %	38.6	30.0
Hypertension, % ^‡^	50.9	41.9
Previous myocardial infarction, %	15.6	6.5
Coronary heart disease, %^§^	53.0	29.6
Congestive heart failure, %^‡^	14.0	6.9
Cerebrovascular disease, %	16.1	8.8
Peripheral vascular disease, %	15.0	5.9
Chronic obstructive pulmonary disease, %^‖^	31.4	22.0
Gastrointestinal ulcer disease, %^‖^	37.3	28.3
Gastrointestinal bleed, %	3.6	2.3
Acute or chronic renal failure, %^‡^	4.0	1.5
Rheumatoid arthritis, %	2.3	1.6
**Concomitant drug treatment**^¶^		
Use of oral corticosteroids, %	4.3	2.1
Use of clopidogrel, %	3.3	1.6
Use of cardioprotective aspirin, %	31.5	22.1

Index date, date of hospitalization with acute MI for cases and matched date for controls; MI, myocardial infarction; NSAID, nonsteroidal anti-inflammatory drug; SD = standard deviation

* Matching variables

^†^ Determined during the time period preceding the index date except where otherwise specified

Ascertained using the date of first occurrence of ICD-9 codes (hospital discharge summary) and by dispensed outpatient medications, including algorithms based on dates of drug dispensing to increase specificity in determining the presence of hypertension, coronary heart disease, congestive heart failure, or rheumatoid arthritis (see Table C in S1 File)

^‡^ Determined only before entry in the cohort since these comorbidities are on the causal pathway between NSAID exposure and the acute MI outcome

^§^ Whereas all hospital diagnosis positions were otherwise considered, for the purpose of a sensitivity analysis, coronary heart disease was more strictly defined and required hospitalization with a diagnosis (ICD-9 code 411.x, 413.x or 414.x) in leading position or codes for percutaneous coronary intervention (480.x) or coronary artery bypass surgery (481.x), or prescriptions defining CHD (algorithm of nitrates, antiplatelet agents, calcium channel blockers, beta-blockers, cardioprotective aspirin and exclusion of other drugs to increase specificity and accuracy of date of first diagnosis) in the 30-day period preceding index date. Prevalence of coronary heart disease by these criteria was 24.2% in cases and 12.5% in controls

^‖^ Comorbidities without any algorithm to overcome the low specificity of drug treatment–chronic pulmonary obstructive disease and gastrointestinal ulcer disease–were ascertained based on dispensed outpatient medications in the one year preceding the index date and based on hospitalization at any time before the index date

^¶^ Determined in the 30-day period preceding the index date

Previous MI and CHD were approximately twice as prevalent for cases as for controls whereas differences in prevalence were more modest for hypertension. How we identified patients with CHD (broader vs stricter criteria) influenced the observed prevalence (in the cases, 53.0% vs 24.2% and in the controls 29.6% vs 12.5%). Use of cardioprotective aspirin, which was defined by a prescription duration overlapping with the index date (Figure B in S1 File) was 31.5% in the cases and 22.1% in the controls.

The model that represents current NSAID exposure by its use on the index date had the overall worst fit based on the AIC whereas representing current NSAID exposure by its daily dose on the index date or any of the 7 prior days had the best fit (Table D in S1 File). A previous nested case-control analysis of NSAID and MI risk in the same RAMQ dataset[14] was based on the latter definition of NSAID exposure. The ORs (95%CI) for current exposure to the most common daily dose versus no current exposure, were: celecoxib 200 mg: 1.16 (1.10, 1.22), diclofenac 150 mg: 1.59 (1.38, 1.84), ibuprofen 1200 mg: 1.42 (1.17, 1.74), naproxen 750 mg: 1.38 (1.21, 1.58), and rofecoxib 25 mg: 1.54 (1.43, 1.66) (see also Table E in S1 File).

In the present investigations of additive interactions, the primary analysis defined current exposure as dose of the NSAID on the index date or any of the 7 prior days while the secondary analysis defined current exposure as use of the NSAID on index date. For diclofenac and ibuprofen there was no evidence of additive joint effects on MI risk with any of the studied CV risk profiles. As shown in Table 2 listing the NSAIDs and CV risk profiles for which we found evidence of a departure from exact additivity (based on RERI and its 95%CI not overlapping with ‘0’), we found additive interactions involving naproxen, celecoxib, and rofecoxib.

**Table 2:** Findings of additive interaction on MI risk between current NSAID use and hypertension or coronary comorbidities–nested case-control analyses of a RAMQ cohort of elderly individuals.

	Relative excess risk due to interaction, RERI (95%CI)*^†^		
	Hypertension	Coronary heart disease	Prior myocardial infarction
Primary analysis–NSAID exposure is current dose on index date or any of the 7 prior days			
**Celecoxib**	— ^‡^	**-0.26 (-0.50, -0.02)**	0.30 (-0.03, 0.62)
**Naproxen**	**-0.28 (-0.52, -0.04)**	—	—
**Rofecoxib**	—	—	—
Secondary analysis–NSAID exposure is current use on index date			
**Celecoxib**	—	-0.20 (-0.48, 0.08)	**0.55 (0.09, 1.01)**
**Naproxen**	**-0.49 (-0.97, -0.02)**	—	—
**Rofecoxib**	—	—	**0.68 (0.04, 1.31)**
*			

* Relative excess risk due to interaction (RERI_OR_) = OR_11_—OR_10_—OR_01_ + 1 or exp(β_10_+ β_01_ + β_11_)—exp(β_10_)—exp(β_01_) + 1. This measures the extent to which, on a difference scale, the effect of both exposures together exceeds the sum of the effects of the two exposures considered separately. Super-additivity (i.e. greater than additive joint effects) means that the number of acute MI cases due to an NSAID in the presence of a CV comorbidity is greater than the sum of the number of MI cases arising independently from either the NSAID exposure or the CV comorbidity. If RERI_OR_ (95%CI) > 0, joint effects are considered to be super-additive. If RERI_OR_ (95%CI) < 0, joint effects are considered sub-additive. Numerical results for RERI_OR_ cannot be used to make statements about the relative magnitude of the underlying additive interactions without knowing how baseline risks differ across groups. However only the direction, rather than the magnitude, of RERI_OR_ is needed to draw conclusions about the public health relevance of interaction.[42]

^†^ Based on conditional logistic regression models for which testing of interactions showed deviations from additive joint effects.

Best-fitting models include product terms for interactions as reported above

Models are adjusted for the following confounders of NSAID-acute MI association: age at index date, diabetes, hyperlipidemia, hypertension, previous myocardial infarction, coronary heart disease, cerebrovascular disease, congestive heart failure, peripheral vascular disease, chronic obstructive pulmonary disease, gastrointestinal ulcer disease, gastrointestinal bleeding, acute or chronic renal failure, and rheumatoid arthritis, concomitant use of oral corticosteroids, clopidogrel, and cardioprotective aspirin. Also adjusted for recent use and past use of celecoxib, diclofenac, ibuprofen, naproxen, rofecoxib, and ‘other NSAIDs, ‘Other NSAIDs’ group composed of diflunisal, etodolac, fenoprofen, flurbiprofen, ketoprofen, indomethacin, mefenamic acid, meloxicam, nabumetone, piroxicam, sulindac, tenoxicam, tiaprofenic acid, tolmetin sodium.

‡ Product term did not enter the best-fitting models. Refer to Table F in S1 File, which gives measures of interaction on the additive and multiplicative scales for each product term tested individually in preliminary models.

The primary and secondary analyses agreed in finding sub-additive effects on MI risk for naproxen and hypertension. In the primary analysis, celecoxib and CHD were sub-additive for MI risk but this could not be shown in the secondary analysis. New interactions uncovered by the secondary analysis (but not documented in the primary analysis) were the super-additive joint effects of previous MI and celecoxib and rofecoxib (Table 2).

Results for NSAIDs and CHD were unchanged when defining coronary heart disease using stricter clinical criteria. Finally, irrespectively of how NSAID exposure was specified, we found exactly additive effects and report no additive interaction between diclofenac, ibuprofen, naproxen, rofecoxib or celecoxib and cardioprotective aspirin (see also Table F in S1 File).

## Discussion

### Summary of findings

Two alternative analyses of additive joint effects found that concomitant use of cardioprotective aspirin does not attenuate the risk of acute MI with celecoxib, rofecoxib, diclofenac, ibuprofen or naproxen. Findings of interaction between NSAIDs and CV comorbidities were not robust to the definition of the NSAID exposure, and for naproxen and hypertension, were even counterintuitive. Indeed we found that joint exposure to hypertension and naproxen (but not the other NSAIDs) was sub-additive for MI. Yet, in investigating these single risk factors, we found that hypertension or exposure to naproxen independently increased risk of acute MI. This sub-additive interaction could be either a spurious finding or the result of competing antagonism [47] such that when hypertension co-occurs with current use of naproxen they compete to trigger acute MI. In this large dataset we were unable to consistently demonstrate interactions between an individual’s pre-existing coronary disease and NSAID associated MI. Indeed, the presence of CHD was or tended to be sub-additive with celecoxib and rofecoxib whereas a previous myocardial infarction suggested a super-additive effect.

### Comparison with other research

Based on the putative model for NSAID toxicity, cardioprotective low-dose aspirin could be expected to mitigate MI risk in NSAID users. Yet, reports of drug-drug interactions due to competition between aspirin and ibuprofen or naproxen for binding to COX-1 enzyme [48–50] have fueled concerns that these NSAIDs may impair aspirin cardioprotection. The PRECISION trial [3] found that celecoxib (209±37 mg) was non-inferior to ibuprofen (2045±246 mg) and naproxen (852±103 mg) for adverse CV events in arthritis patients at moderate CV risk.[3, 51] These conclusions have been challenged namely with the assertion that celecoxib does not interact with cardioprotective aspirin, thus potentially biasing the trial results against ibuprofen and naproxen.[52, 53] Intuitively, cardioprotective aspirin could be predicted to mitigate the risk of MI in NSAID users. However, NSAIDs and aspirin share a common docking site on COX-1, raising the potential for a competitive interaction between these drugs. [54] In vivo [48, 50, 54–57], ex vitro [49, 58], and in vitro [58] investigations agree that ibuprofen and naproxen interfere with the pharmacodynamic effects of aspirin, and that diclofenac does not. Evidence of a pharmacodynamic interaction of aspirin with rofecoxib is apparently limited to a single in vivo study. [55] Data on celecoxib are conflicting. Studies in healthy volunteers [48, 50] suggest there is no interference by celecoxib on aspirin effect on platelets while all [58–60] but one [49] ex vivo study found that celecoxib interfered with aspirin’s action on COX-1. Differences in experimental conditions [59] or method of platelet function testing [61] may explain why results of pharmacodynamic studies might be discordant. A review of this literature concludes that celecoxib can attenuate the antiplatelet effects of low-dose aspirin in a dose-dependent fashion.[62] Our findings that NSAIDs and cardioprotective aspirin have an exactly additive effect on acute MI suggest that pharmacological studies of interaction between these drugs might have an imperfect clinical correlate. Alternatively, the extent to which aspirin and individual NSAIDS interact with regard to risk of MI might vary, and this potential interaction might also be influenced by residual confounding. Moreover underascertainment of OTC cardioprotective aspirin use may have occurred.

Previous registry [63] and database studies [64, 65] found no significant interactions on MI risk between NSAIDs and cardioprotective aspirin or hypertension. To our knowledge, there are only two previous studies of NSAIDs and CV profile interactions on the additive scale. Garcia Rodriguez [66] gave RERI point estimates in reference to acute MI with ibuprofen or naproxen in those also taking aspirin but without the associated confidence limits, which precludes a full interpretation of their results. Solomon and colleagues [67] investigated additive interactions using a measure of Rothman’s proportion attributable to interaction (AP or RERI_RR_/RR_11_) [42] but the outcome was a composite cardiovascular endpoint and not limited to MI.

### Strengths and limitations

In this study, misclassification was minimized by measuring the status of exposures and confounders for each day of follow-up time. Confounding was controlled by a multivariable regression, and matching on baseline characteristics, date of cohort entry, and duration of follow-up time in the cohort. This matching on time is an important quality feature. Although rofecoxib has been off the market for over a decade it is still relevant to discussing the CV safety of NSAIDs. It is worth emphasizing that choosing a timeframe before rofecoxib withdrawal is the most effective way to minimize the influence of confounding by indication or by contraindication and of selective prescribing in an observational study of NSAIDs CV safety. Another strength of our work is the prespecified alternative specifications of NSAID exposure-related variables. With such approach, we uncovered conflicting results for interaction between NSAID-associated MI risk and pre-existing coronary disease.

In this healthcare database study, exposure misclassification due to ‘as needed’ use of NSAIDs may be expected to have occurred in a non-differential manner between cases and controls. Underascertainment of OTC ibuprofen and cardioprotective aspirin use is possible although the older adults enrolled in this study likely sought a prescription to be reimbursed for the cost of these medications.[14, 68] Whereas exposure to ibuprofen may have been underestimated due to OTC use, especially for short-term low doses, it may have been overestimated when ibuprofen was prescribed ‘as needed’.[14] Silent MIs or out-of-hospital fatal MIs are not captured in the source database however we do not expect that these would differ from documented MI by exposure to NSAIDs.

As we anticipated when designing this study, uncontrolled biases can spuriously mask or suggest the presence of additive interactions. Therefore, alternative explanations for our results include systematic and random errors[7]. The finding of a sub-additive interaction involving hypertension and naproxen, but not the other NSAIDs, is suspicious. If competing antagonism is an explanation for the naproxen-hypertension interaction then this would also be expected to occur for other interaction pairs. Likewise the results from the primary analysis and the secondary analysis would not be expected to differ if competing antagonism was the explanatory mechanism. Similar to other NSAIDs, the effect of naproxen on acute MI risk may involve mediation[69] by hypertension (or increased blood pressure).[70–72] A cohort study reported observing a dose response between naproxen and systolic blood pressure.[73] As our study was solely based on administrative health data, the association of increased blood pressure with acute MI could not be controlled for the confounding effect of prior naproxen use and of previous blood pressure values over time (see Figure D in S1 File). Residual confounding of naproxen-associated MI by elevated blood pressure may or may not explain why hypertension and naproxen were sub-additive.

The presence of CHD is both a potentially interacting CV risk factor and a potential confounder of the celecoxib-acute MI association (Figure E, panel 1 in S1 File). Smoking data was unavailable and consequently, the association between existing coronary heart disease and NSAID associated acute MI may be confounded by smoking thereby leading to a biased RERI[74] (see Figure E, panel 2 in S1 File). This is an alternative explanation for the finding of a sub-additive effect between celecoxib and CHD. Assuming there is possible residual confounding of CHD by smoking, if the distribution of CHD in the population is not independent of celecoxib prescriptions and if smoking interacts with celecoxib on the additive scale (see Figure E, panel 2 in S1 File), then RERI may be biased. [74] Although it has been proposed to perform sensitivity analysis of interaction estimates by using bias corrected formulae,[75] implementation in this study would be problematic since we are possibly dealing with multiple CV risk profiles and multiple NSAID exposures for which bias correction might be needed.

As with all database studies, which measure drug dispensing and not actual drug intake, it was impossible to perfectly capture real-life adherence. Moreover, ascertainment of CV risk profiles is not immune to misclassification. Bias corrected formulae for sensitivity analysis of interaction estimates under measurement error [74] assume that misclassification of NSAID occurs independently of misclassification of the CV risk profile and that it is non-differential between cases and controls, which may not always be true. Also, type I error could have occurred due to multiple testing.

In general, fairly large sample sizes are required to detect interaction, [76] and the power to detect statistical interactions is said to be typically an order of magnitude less than the power to detect main effects. [77] Despite the large size and the common prevalence of CV risk profiles in this study (Table 1), current NSAID exposure may have been too sparse, resulting in a very small effective sample potentially unsuitable for interaction studies. [78] Lastly, being able to conclude about deviation from exact additivity of risks hinges on the precision of the estimate for RERI. In some instances its confidence interval was very wide indicating that power was indeed limited to make definitive conclusions. In such case, the more appropriate interpretation is that RERI was inconclusive. In future studies, Bayesian estimation might be helpful in that regard. [79] The posterior distribution could provide the probability that a RERI is greater than zero, thus allowing additional inferences about additive interactions.

## Conclusion

Assessing interaction on the additive scale is a relatively new practice in pharmacoepidemiologic studies. Despite–or maybe perhaps of methodological rigour–we were unable to demonstrate consistent interactions between an individual’s cardiovascular comorbidities and NSAID-associated acute MI. Our study highlights challenges for studying multiple two-way additive interactions in a healthcare database. A key substantive finding, supported by sensitivity analyses, is that cardioprotective aspirin does not seem to mitigate the risk of acute MI for any of the common NSAIDs. Replication of these results is nonetheless needed as are further studies of additive joint effects on MI for NSAIDs and hypertension or pre-existing coronary disease. Our work underscores the need for verifying the robustness of additive interaction findings.

## Supporting information

S1 File Supporting information tables and figures. (DOCX)
