# Supplementary material for: Studying additive interaction in a healthcare database: Case study of NSAIDs, cardiovascular profiles, and acute myocardial infarction
Source: PLoS One. 2018 Aug 10;13(8):e0201884. doi: 10.1371/journal.pone.0201884 (PMC6086415; doi:10.1371/journal.pone.0201884)
Supplement: S1 File — (DOCX) [file pone.0201884.s001.docx]

**S1 –Supporting Information – Studying additive interaction in a healthcare database: case study of NSAIDs, cardiovascular profiles, and acute myocardial infarction**

1. Creation of episodes of use given various scenarios for consecutive prescriptions of the same NSAID in nested case-control dataset sourced from RAMQ

| **NSAID prescription scenario ^*^** | **Illustration using celecoxib as example** | **Decision for creating episodes of continuous use** |
| --- | --- | --- |
| Second prescription starts and ends after the first one  No overlap of coverage dates  Lag between the two prescriptions ≤ 7 days  Same daily dose | 14 days  7 days  14 days  Lag  Celecoxib 200 mg/day  Celecoxib 200 mg/day | Extend episode of use  35 days  Celecoxib 200 mg/day |
| Second prescription starts and ends after the first one  No overlap of coverage dates  Lag between the two prescriptions ≤ 7 days  Different daily dose | Celecoxib 100 mg/day  Lag  14 days  7 days  14 days  Celecoxib 200 mg/day | Start a new episode of use  Celecoxib 200 mg/day  Celecoxib 100 mg/day  14 days  14 days |
| Second prescription starts and ends after the first one  No overlap of coverage dates  Lag between the two prescriptions > 7 days  Same or different daily dose | Celecoxib 200 mg/day  Celecoxib 200 mg/day  Lag  14 days  14 days  14 days | Start a new episode of use  Celecoxib 200 mg/day  Celecoxib 200 mg/day  14 days  14 days |
| Second prescription starts and ends after the first one  Overlap of coverage dates  Same daily dose | 14 days  14 days  Celecoxib 200 mg/day  Celecoxib 200 mg/day | Retain first prescription up to start date of the second one then retain the second prescription  23 days  Celecoxib 200 mg/day |
| Second prescription within coverage dates of the first one  Same daily dose | Celecoxib 200 mg/day  14 days  Celecoxib 200 mg/day  7 days | Retain only the first prescription  Celecoxib 200 mg/day  14 days |
| Second prescription within coverage dates of the first one  Different daily dose  First prescription has higher daily dose | Celecoxib 100 mg/day  Celecoxib 200 mg/day  14 days  7 days | Retain the prescription with the highest daily dose, therefore retain the first prescription  Celecoxib 200 mg/day  14 days |
| Second prescription within coverage dates of the first one  Different daily dose  Second prescription has higher daily dose | Celecoxib 100 mg/day  Celecoxib 200 mg/day  14 days  7 days | Select the prescription with the highest daily dose, therefore retain the first prescription up to start date of the second one  Celecoxib 100 mg/day then 200 mg/day  10 days |
| First and second prescription have same start date but different end date  Different daily dose | Celecoxib 100 mg/day  Celecoxib 200 mg/day  14 days  21 days | Retain the prescription with highest daily dose  Celecoxib 200 mg/day  14 days |
| First and second prescription have same start date but different end date  Same daily dose | Celecoxib 200 mg/day  14 days  Celecoxib 200 mg/day  21 days | Retain the prescription with longest duration  21 days  Celecoxib 200 mg/day |
| First and second prescription have same start date and same end date  Different daily dose | Celecoxib 200 mg/day  Celecoxib 100 mg/day  14 days  14 days | Retain the prescription with the highest daily dose (if summing up dose, daily dosage may exceed usual range  14 days  Celecoxib 200 mg/day |
| First and second prescription have same start date and same end date  Same daily dose | Celecoxib 200 mg/day  Celecoxib 200 mg/day  14 days  14 days | Retain the second prescription  14 days  Celecoxib 200 mg/day |
| **^*^** When building NSAID episodes, usage on an ‘as needed’ basis was estimated by reconciling daily doses that are used in clinical practice and dispensing data for each drug fill. | | |

1. Creation of episodes of use given various scenarios for consecutive prescriptions of two different NSAIDs in nested case-control dataset sourced from RAMQ

| **NSAID prescription scenario ^*^** | **Illustration using celecoxib and rofecoxib as examples** | **Decision for creating episodes of use** |
| --- | --- | --- |
| Second prescription starts and ends after the first one  No overlap of coverage dates  Different NSAIDs | 14 days  14 days  Celecoxib 200 mg/day  Rofecoxib 25 mg/day | Start a new episode of use  Celecoxib 200 mg/day  Rofecoxib 25 mg/day  14 days  14 days |
| Second prescription starts and ends after the first one  Overlap of coverage dates  Different NSAIDs | 14 days  14 days  Celecoxib 200 mg/day  Rofecoxib 25 mg/day | Start a new episode of use at the start date of the second prescription  Celecoxib 200 mg/day  Rofecoxib 25 mg/day  14 days  10 days |
| Second prescription within coverage dates of the first one  Different NSAIDs | Rofecoxib 25 mg/day  Celecoxib 200 mg/day  14 days  7 days | Start a new episode of use at the start date of the second prescription  Celecoxib 200 mg/day  Rofecoxib 25 mg/day  7 days  3 days |
| Two prescriptions have same start date but different end date  Different NSAIDs | Rofecoxib 100 mg/day  Celecoxib 200 mg/day  14 days  21 days | Retain the prescription with longest duration  21 days  Rofecoxib 25 mg/day |
| Two prescriptions have same start date and same end date  Different NSAIDs | Rofecoxib 25 mg/day  Celecoxib 200 mg/day  14 days  14 days | Select at random the prescription to retain  14 days  Celecoxib 200 mg/day |
| **^*^** When building NSAID episodes, usage on an ‘as needed’ basis was estimated by reconciling daily doses that are used in clinical practice and dispensing data for each drug fill | | |


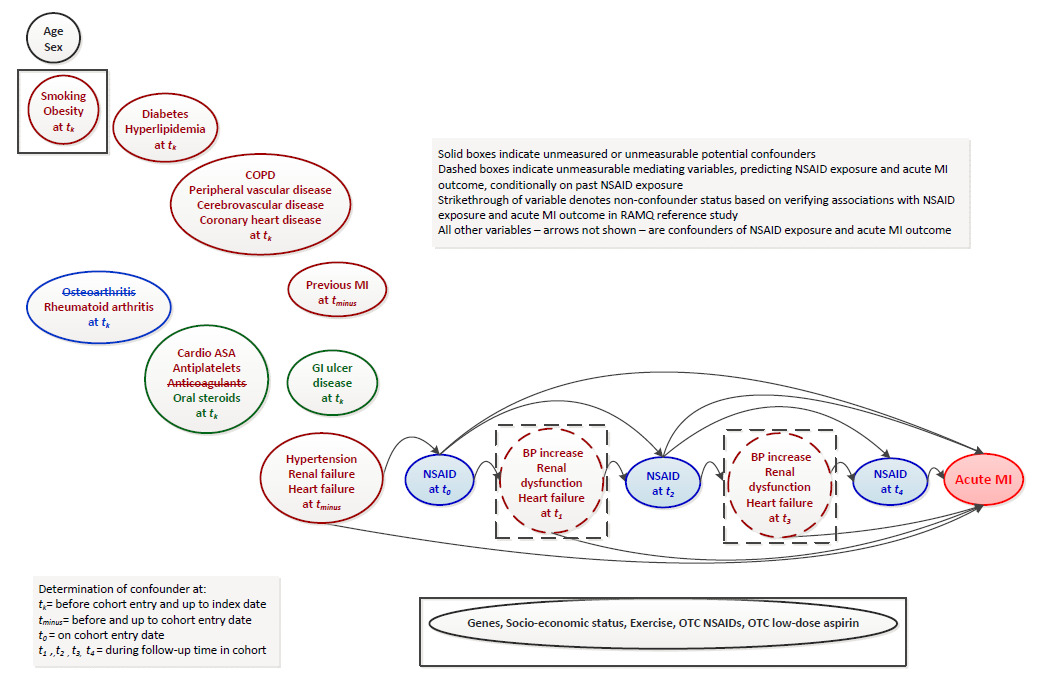


1. Identification of confounders of NSAID exposures and acute MI outcome showing time-dependent confounding affected by previous exposure
   We substantiated confounder status for each candidate covariate, by calculating the odds ratio (OR) of association between the covariate and exposure to NSAIDs among controls and the OR of association between the covariate and MI outcome in the unexposed, leading to the final set of confounders selected for adjustment (see S1 Table C)
2. Definition and ascertainment of comorbidities and concomitant drugs in nested case-control dataset sourced from RAMQ

| **Confounder** **^*^** | **Definition via hospitalizations based on ICD-9 codes** ^†^ | **Definition via outpatient prescriptions**  **Drugs or drug classes or drug algorithms** ^‡^ | | **Validation study or guideline** ^§^ **Positive predictive value – Hospital discharge database  (95% confidence interval)** ^‖^ | | **Determination period** ^¶^ | |
| --- | --- | --- | --- | --- | --- | --- | --- |
| **Comorbidities** |  |  | |  | |  | |
| Diabetes | 250 | Metformin, sulfonylureas, thiazolidinediones, meglitinides, acarbose, insulins, insulin analogues, insulin administration materials, blood glucose test reagents | | With complications 90.5 (82.1-95.8) Without complications 92.6 (89.7-94.9) | | Over time preceding index date | |
| Hyperlipidemia | 272.0-272.5 , 272.7-272.9 | Statins, fibrates, niacin, resins, probucol, ezetimibe | | -- | | Over time preceding index date | |
| Hypertension | 401, 402, 403, 404, 405 | Algorithm of diuretics, potassium-sparing agents, beta-blockers, calcium channel blockers, angiotensin-converting enzyme inhibitors, angiotensin receptor blockers, alpha blockers, sympatholytics  Exclusion of other drugs to increase specificity (blood-pressure-lowering drugs also used for treating other comorbidities) and increase accuracy of date of first diagnosis | | 97.7 (96.5-98.5) | | Before cohort entry | |
| Previous myocardial infarction ^a^ | 410, 412 | Not defined by prescriptions due to low specificity of drug treatment | | 94.1 (90.4-96.8) | | Over time preceding index date | |
| Coronary heart disease ^b^ | 411, 413, 414  Procedures: 480, 481 | Algorithm of nitrates, antiplatelet agents, calcium channel blockers, beta-blockers, cardioprotective aspirin  Exclusion of other drugs to increase specificity and accuracy of date of first diagnosis | | -- | | Over time preceding index date | |
| Congestive heart failure | 428, 429 | Algorithm of loop diuretics, spironolactone, beta-blockers, angiotensin-converting enzyme inhibitors, angiotensin receptor blockers, hydralazine, nitrates, digoxin  Exclusion of other drugs to increase specificity and accuracy of date of first diagnosis | | Liu 2003 Lee 2004 Arnold 2006 Saczynski 2012  79.6 (76.1-83.2) | | Before cohort entry | |
| Cerebrovascular disease | 430, 431, 432, 433, 434, 435, 436, 437, 438 | Nimodipine | Roumie 2008 Andrade 2012  68.9 (62.4-75.5) | | Over time preceding index date | |  |
| Peripheral vascular disease | 440, 443.8, 443.9, 444.2 | Pentoxifylline | AHIMA 2009  95.0 (91.3-97.5) | | Over time preceding index date | |  |
| Chronic obstructive pulmonary disease | 491, 492, 496 | Algorithm of xanthines, inhaled beta-2 agonists, inhaled corticosteroids, inhaled anticholinergics | Cooke 2011  94.9 (91.6-97.2) | | Over time preceding index date | |  |
| Gastrointestinal ulcer disease | 531.1, 531.3, 531.5, 531.7, 531.9, 532.1, 532.3, 532.5, 532.7, 532.9, 533.1, 533.3, 533.5, 533.7, 533.9, 534.1, 534.3, 534.5, 534.7, 534.9, 535.0, 535.1, 535.5, 535.6, 536.8 | Algorithm of H2-receptor blockers, proton pump inhibitors, misoprostol, pirenzepine, sucralfate | Abraham 2006 | | Over time preceding index date | |  |
| Gastrointestinal bleed | 531.0, 531.2, 531.4, 531.6, 532.0, 532.2, 532.4, 532.6, 533.0, 533.2, 533.4, 533.6, 534.0, 534.2, 534.4, 534.6, 578, 535.01, 535.51, 535.61 | Not defined by prescriptions due to low specificity of drug treatment | Abraham 2006 | | Over time preceding index date | |  |
| Acute or chronic renal failure | 584, 585, 586 | Algorithm of erythropoietins, sevelamer | --  Acute renal disease  83.6 (75.4-90.0)  Chronic renal disease  96.1 (93.1-98.0) | | Before cohort entry | |  |
| Rheumatoid arthritis | 714 | Algorithm of methotrexate, disease-modifying anti-rheumatic drugs, gold salts, corticosteroids, biologic agents | Tavares 2011 | | Over time preceding index date | |  |
| **Concomitant drugs** |  |  |  | |  | |  |
| Oral corticosteroids | -- | Betamethasone, budesonide, cortisone acetate, dexamethasone, hydrocortisone, methylprednisolone, prednisolone, prednisone, triamcinolone | -- | | In the 30 days preceding index date | |  |
| Clopidogrel | -- | Clopidogrel | -- | | In the 30 days preceding index date | |  |
| Cardioprotective aspirin | -- | Aspirin 80 mg every other day to 650 mg daily | Pignone 2010 | | In the 30 days preceding index date | |  |
| ^--^ Not applicable or not available  ^*^ Ascertained by date of first occurrence of hospital diagnostic codes or outpatient prescription drugs or both  ^†^ In primary diagnosis position or any of 15 secondary diagnosis positions  ^‡^ Using drugs available for treating comorbidity during study period  ^§^ Published validation studies and treatment guidelines applicable during study period supporting definition: Liu P, Arnold JM, Belenkie I, et al. The 2002/3 Canadian Cardiovascular Society consensus guideline update for the diagnosis and management of heart failure. Can J Cardiol. 2003;19(4):347-56.  Lee DS, Mamdani MM, Austin PC, et al. Trends in heart failure outcomes and pharmacotherapy: 1992 to 2000. Am J Med. 2004;116(9):581-9.  Arnold JM, Liu P, Demers C, et al. Canadian Cardiovascular Society consensus conference recommendations on heart failure 2006: diagnosis and management. Can J Cardiol. 2006;22(1):23-45.  Saczynski JS, Andrade SE, Harrold LR, et al. A systematic review of validated methods for identifying heart failure using administrative data. Pharmacoepidemiol Drug Saf. 2012;21:129-40.  Roumie CL, Mitchel E, Gideon PS, Varas-Lorenzo C, Castellsague J, Griffin MR. Validation of ICD-9 codes with a high positive predictive value for incident strokes resulting in hospitalization using Medicaid health data. Pharmacoepidemiol Drug Saf. 2008;17(1):20-6.  Andrade SE, Harrold LR, Tjia J, et al. A systematic review of validated methods for identifying cerebrovascular accident or transient ischemic attack using administrative data. Pharmacoepidemiol Drug Saf. 2012;21:100-28.  American Health Information Management Association. Coding for peripheral vascular disease (PVD). Audio Seminar/Webinar August 20, 2009. [http://campus.ahima.org/audio/2009/RB082009.pdf. Last accessed June 10](http://campus.ahima.org/audio/2009/RB082009.pdf.%20Last%20accessed%20June%2010), 2018.  Cooke CR, Joo MJ, Anderson SM, et al. The validity of using ICD-9 codes and pharmacy records to identify patients with chronic obstructive pulmonary disease. BMC Health Serv Res. 2011;11:37.  Abraham NS, Cohen DC, Rivers B, Richardson P. Validation of administrative data used for the diagnosis of upper gastrointestinal events following nonsteroidal anti-inflammatory drug prescription. Aliment Pharmacol Ther. 2006;24(2):299-306.  Tavares R, Pope JE, Tremblay JL, et al. Early management of newly diagnosed rheumatoid arthritis by Canadian rheumatologists: a national, multicenter, retrospective cohort. J Rheumatol. 2011;38(11):2342-5.  Pignone M, Alberts MJ, Colwell JA, et al. Aspirin for primary prevention of cardiovascular events in people with diabetes. J Am Coll Cardiol. 2010 6/22/;55(25):2878-86.  ^‖^ Lambert L, Blais C, Hamel D, Brown K, Rinfret S, Cartier R, et al. Evaluation of care and surveillance of cardiovascular disease: can we trust medico-administrative hospital data? Can J Cardiol. 2012;28(2):162-8.  ^¶^ Determined using the date of first occurrence of ICD-9 codes (hospital discharge summary) and by dispensed outpatient medications Hypertension, congestive heart failure, and renal failure were ascertained only before entry in the cohort since these comordbities are on the causal pathway between NSAID exposure and the acute MI outcome Comorbidities without any algorithm to overcome the low specificity of drug treatment – chronic pulmonary obstructive disease and gastrointestinal ulcer disease – were ascertained based on dispensed outpatient medications in the one year preceding the index date (date of hospitalization with acute myocardial infarction for cases and matched date for controls) and based on hospitalization at any time before the index date  ^a^ Determined over the period preceding index date (recorded before the acute MI case definition) to account for possible under determination on hospital discharge summary  ^b^ Whereas all hospital diagnosis positions were otherwise considered, for the purpose of pilot work, coronary heart disease was more strictly defined and required hospitalization with ICD-9 code 411.x, 413.x or 414.x in leading diagnosis position or codes for percutaneous coronary intervention (480.x) or coronary artery bypass surgery (481.x) , or prescriptions defining CHD (algorithm of nitrates, antiplatelet agents, calcium channel blockers, beta-blockers, cardioprotective aspirin and exclusion of other drugs to increase specificity and accuracy of date of first diagnosis) in the 30-day period preceding index date | | | | | | |  |


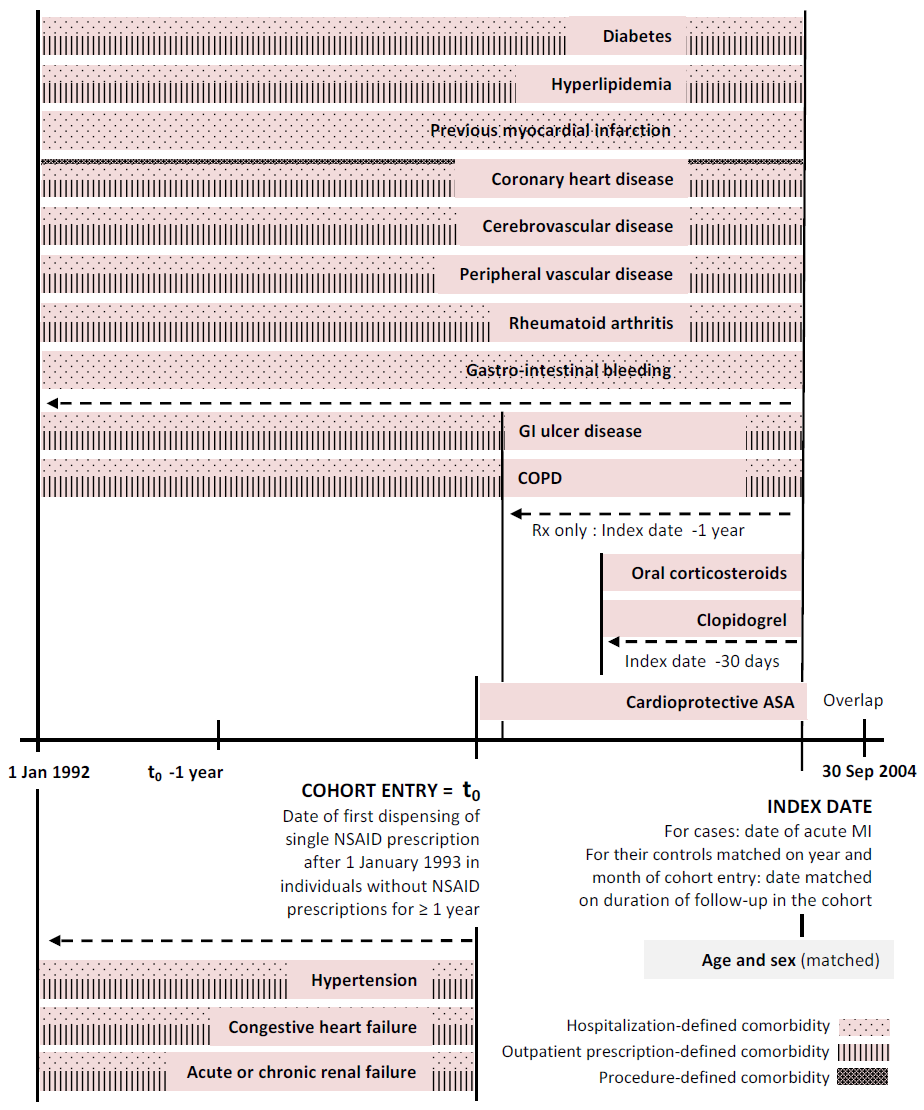


1. Determination of comorbidities and concomitant drug treatments in nested case-control dataset sourced from RAMQ


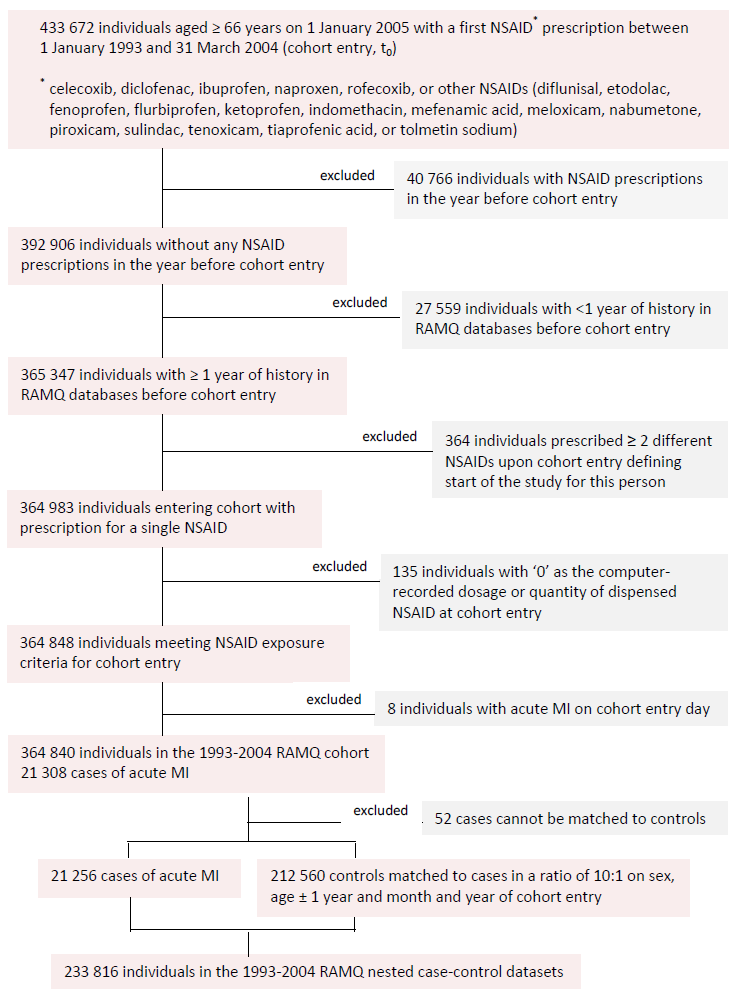


1. Subject flow in nested case-control dataset sourced from RAMQ
2. Fit to the data of nested case-control models of the effect of NSAIDs on the risk of acute MI

| **Type of model** | **AIC**^*^ |
| --- | --- |
| **Nested case-control models where NSAID current exposure to each NSAID was modelled as:** |  |
| Current use (binary indicator) on index date^†^ | 93865.9 |
| Current use (binary indicator) on index date or any of the 7 prior days^‡^ | 93844.9 |
| Current dose (continuous variable) on index date^†^ | 93852.2 |
| Current dose (continuous variable) on index date any of the 7 prior days^‡^ | 93834.6 |
| AIC, Akaike information criterion; Index date, date of hospitalization with acute myocardial infarction for cases and matched date for controls; MI, myocardial infarction; NSAID, nonsteroidal anti-inflammatory drug; NCC, nested case-control  ^*^ See Table 1 for description of adjustments  ^†^ Recent and past exposures to each NSAID were represented by mutually exclusive binary indicators, where recent use= 1-30 days before the index date, and past use= 31-180 days before the index date  ^‡^ Recent and past exposures to each NSAID were represented by mutually exclusive binary indicators, where recent use= 8-30 days before the index date, and past use= 31-180 days before the index date | |

1. Adjusted ORs (with 95% CI) for the association between MI risk and NSAID exposure in a nested case-control analysis of a RAMQ cohort of elderly individuals

| **NSAID exposure**^*†^ | **OR** [**95%CI**] ^‡‖^ |
| --- | --- |
| **Celecoxib** |  |
| Past use | 1.07 [1.00-1.15] |
| Recent use | 1.26 [1.12-1.41] |
| Current 100 mg/day | 1.08 [1.05-1.10] |
| Current 200 mg/day | 1.16 [1.10-1.22] |
| Current 400 mg/day | 1.34 [1.21-1.48] |
| **Diclofenac** |  |
| Past use | 1.12 [1.03-1.22] |
| Recent use | 1.29 [1.10-1.51] |
| Current 75 mg/day | 1.26 [1.17-1.36] |
| Current 100 mg/day | 1.36 [1.24-1.50] |
| Current 150 mg/day | 1.59 [1.38-1.84] |
| **Ibuprofen** |  |
| Past use | 1.13 [0.98-1.30] |
| Recent use | 1.30 [0.98-1.72] |
| Current 600 mg/day | 1.19 [1.08-1.32] |
| Current 1200 mg/day | 1.42 [1.17-1.74] |
| Current 1800 mg/day | 1.70 [1.26-2.29] |
| **Naproxen** |  |
| Past use | 1.15 [1.04-1.26] |
| Recent use | 1.28 [1.06-1.54] |
| Current 500 mg/day | 1.24 [1.14-1.36] |
| Current 750 mg/day | 1.38 [1.21-1.58] |
| Current 1000 mg/day | 1.54 [1.29-1.84] |
| **Rofecoxib** |  |
| Past use | 1.07 [0.99-1.15] |
| Recent use | 1.12 [0.98-1.29] |
| Current 12.5 mg/day | 1.24 [1.20-1.29] |
| Current 25 mg/day | 1.54 [1.43-1.66] |
| Current 50 mg/day | 2.38 [2.05-2.76] |
| CI, confidence interval; OR, odds ratio; MI, myocardial infarction; NSAID(s), nonsteroidal anti-inflammatory drug(s).  ^*^ For each NSAID use was characterized as: current daily dose= dose of a prescription supply that covered the index date or any of the 7 days before; recent use= duration of prescription supply ended 8-30 days before the index date; past use= duration of prescription supply ended 31-180 days before the index date; non-use= no use in the 180 days preceding the index date For a given NSAID, ‘current’, ‘recent’, ‘past’ use and ‘non-use’ categories are mutually exclusive  ^†^ A current dose could have been taken for any duration. Past use (31-180 days ago) or recent use (8-30 days ago) could be for any dose and any duration within the time period  ^‡^ The reference for ORs for past and recent uses for a given NSAID was set to non-use in the last 180 days of this NSAID. Current dose was modeled a single continuous variable and ORs were calculated for different typical doses by multiplying the parameter estimate by the doses specified. The OR for a given current dose estimates the change in the MI risk associated with a corresponding increase in the current daily dose, and compares, for example, the current daily dose indicated versus no current exposure (0 mg/day).  ^§^ Adjusted for current daily dose of each NSAID in this Table and for recent and past use of each NSAID in this Table; also adjusted for current, recent, and past use of other prescription NSAIDs grouped as ‘other NSAIDs’ (diflunisal, etodolac, fenoprofen, flurbiprofen, ketoprofen, indomethacin, mefenamic acid, meloxicam, nabumetone, piroxicam, sulindac, tenoxicam, tiaprofenic acid, tolmetin sodium)  ^‖^ Adjusted for age at index date, diabetes, hyperlipidemia, hypertension, previous myocardial infarction, coronary heart disease, cerebrovascular disease, congestive heart failure, peripheral vascular disease, chronic obstructive pulmonary disease, gastrointestinal ulcer disease, gastrointestinal bleeding, acute or chronic renal failure, and rheumatoid arthritis, concomitant use of oral corticosteroids, clopidogrel, and cardioprotective aspirin | |

1. Measures of interaction on the additive and multiplicative scales between each CV risk profile and each current NSAID in the primary and secondary analyses in a nested case-control analysis of a RAMQ cohort of elderly individuals – Each product term tested individually

| **Cardiovascular risk factor** | **Primary analysis – NSAID exposure is current dose on index date or any of the 7 prior days** | | | **Secondary analysis – NSAID exposure is current use on index date** | | |
| --- | --- | --- | --- | --- | --- | --- |
|  | Base model^*^ +  each product term added one at a time  Wald test | RERI (95%CI)^†^ | exp(β_11_)^‡^ | Base model^*^ +  each product term added one at a time  Wald test | RERI (95%CI) | exp(β_11_) |
| **Cardioprotective aspirin** | | | | | | |
| celXasa | p=0.8018 | -0.02 (-0.16, 0.13) | 0.98 (0.85, 1.12) | p=0.4585 | -0.05 (-0.24, 0.13) | 0.94 (0.80, 1.09) |
| dicXasa | p=0.6449 | -0.07 (-0.35, 0.21) | 0.94 (0.68, 1.20) | p=0.9101 | 0.07 (-0.42, 0.55) | 1.02 (0.71, 1.33) |
| ibuXasa | p=0.7503 | 0.10 (-0.56, 0.77) | 1.09 (0.48, 1.70) | p=0.7158 | 0.24 (-0.87, 1.35) | 1.13 (0.40, 1.86) |
| napXasa | p=0.3631 | -0.19 (-0.67, 0.28) | 0.85 (0.56, 1.14) | p=0.3500 | -0.23 (-0.76, 0.30) | 0.83 (0.52, 1.15) |
| rofXasa | p=0.5683 | -0.05 (-0.21, 0.11) | 0.96 (0.81, 1.10) | p=0.2290 | -0.11 (-0.37, 0.14) | 0.90 (0.75, 1.05) |
| **Hypertension** | | | | | | |
| celXht | p=0.3923 | -0.06 (-0.20, 0.08) | 0.95 (0.83, 1.07) | p=0.2621 | -0.08 (-0.26, 0.11) | 0.92 (0.79, 1.05) |
| dicXht | p=0.4637 | -0.09 (-0.33, 0.15) | 0.92 (0.71, 1.13) | p=0.1056 | -0.27 (-0.68, 0.14) | 0.80 (0.59, 1.01) |
| ibuXht | p=0.7037 | 0.10 (-0.44, 0.63) | 1.09 (0.62, 1.55) | p=0.1630 | 0.66 (-0.20, 1.52) | 1.45 (0.69, 2.21) |
| napXht | p=0.0216 | -0.43 (-0.85, -0.01) | 0.72 (0.52, 0.92) | p=0.0212 | -0.49 (-0.96, -0.02) | 0.70 (0.48, 0.91) |
| rofXht | p=0.5306 | 0.05 (-0.11, 0.22) | 1.04 (0.90, 1.19) | p=0.9080 | 0.09 (-0.16, 0.34) | 1.01 (0.85, 1.16) |
| **Coronary heart disease** | | | | | | |
| celXchd | p=0.1911 | -0.16 (-0.38, 0.07) | 0.92 (0.81, 1.03) | p=0.0602 | -0.04 (-0.30, 0.22) | 0.87 (0.74, 1.00) |
| dicXchd | p=0.4478 | -0.17 (-0.59, 0.26) | 0.91 (0.70, 1.13) | p=0.4496 | 0.22 (-0.40, 0.84) | 0.90 (0.66, 1.14) |
| ibuXchd | p=0.5898 | 0.26 (-0.74, 1.26) | 1.13 (0.62, 1.64) | p=0.2219 | 1.28 (-0.20, 2.77) | 1.39 (0.65, 2.13) |
| napXchd | p=0.2367 | 0.05 (-0.56, 0.66) | 0.84 (0.61, 1.08) | p=0.4469 | 0.17 (-0.53, 0.87) | 0.89 (0.61, 1.16) |
| rofXchd | p=0.1059 | -0.19 (-0.44, 0.05) | 0.89 (0.77, 1.02) | p=0.0208 | 0.11 (-0.24, 0.46) | 0.83 (0.71, 0.96) |
| **Previous myocardial infarction** | | | | | | |
| celXmi | p=0.1971 | 0.17 (-0.10, 0.44) | 1.13 (0.92, 1.34) | p=0.1927 | 0.27 (-0.06, 0.60) | 1.14 (0.92, 1.36) |
| dicXmi | p=0.7107 | -0.09 (-0.57, 0.38) | 0.93 (0.57, 1.29) | p=0.4944 | -0.11 (-0.83, 0.61) | 0.86 (0.49, 1.23) |
| ibuXmi | p=0.3806 | -0.39 (-1.11, 0.34) | 0.71 (0.16, 1.25) | p=0.3723 | -0.53 (-1.80, 0.74) | 0.65 (0.04, 1.26) |
| napXmi | p=0.8256 | 0.06 (-0.68, 0.80) | 0.95 (0.56, 1.35) | p=0.7699 | 0.02 (-0.81, 0.85) | 0.93 (0.49, 1.37) |
| rofXmi | p=0.8921 | 0.02 (-0.25, 0.30) | 1.01 (0.81, 1.22) | p=0.6036 | 0.29 (-0.16, 0.73) | 1.06 (0.83, 1.29) |
| asa=cardioprotective aspirin; cel=celecoxib; chd=coronary heart disease dic=diclofenac; ht=hypertension; ibu=ibuprofen; nap=naproxen; mi=history of previous myocardial infarction; RERI= relative excess risk due to interaction; rof=rofecoxib  ^*^ Models for current exposure to celecoxib, diclofenac, ibuprofen, naproxen, and rofecoxib. Adjusted for the following confounders of NSAID-acute MI association: age at index date, diabetes, hyperlipidemia, hypertension, previous myocardial infarction, coronary heart disease, cerebrovascular disease, congestive heart failure, peripheral vascular disease, chronic obstructive pulmonary disease, gastrointestinal ulcer disease, gastrointestinal bleeding, acute or chronic renal failure, and rheumatoid arthritis, concomitant use of oral corticosteroids, clopidogrel, and cardioprotective aspirin. Also adjusted for recent use and past use of celecoxib, diclofenac, ibuprofen, naproxen, rofecoxib, and ‘other NSAIDs’. ‘Other NSAIDs’ group composed of diflunisal, etodolac, fenoprofen, flurbiprofen, ketoprofen, indomethacin, mefenamic acid, meloxicam, nabumetone, piroxicam, sulindac, tenoxicam, tiaprofenic acid, tolmetin sodium  ^†^ Interaction on the additive scale – Relative excess risk due to interaction (RERI_OR_)= OR_11_ − OR_10_ − OR_01_ + 1 or exp(β_10_+ β_01_ + β_11_) - exp(β_10_) - exp(β_01_) + 1  Measures the extent to which, on a difference scale, the effect of both exposures together exceeds the sum of the effects of the two exposures considered separately  If RERI_OR_ (95%CI) > 0, joint effects are greater than additive. If RERI_OR_ (95%CI) < 0, joint effects are less than additive  ^‡^ Interaction on the multiplicative scale – Ratio of ORs= OR_11_/ OR_10_ * OR_01_ or exp(β_11_)  Measures the extent to which, on a ratio scale the effect of both exposures together exceeds the product of the effects of the two exposures considered separately  If ratio of ORs (95%CI) > 1, joint effects are greater than multiplicative. If ratio of ORs (95%CI) < 1, joint effects are less than multiplicative | | | | | | |


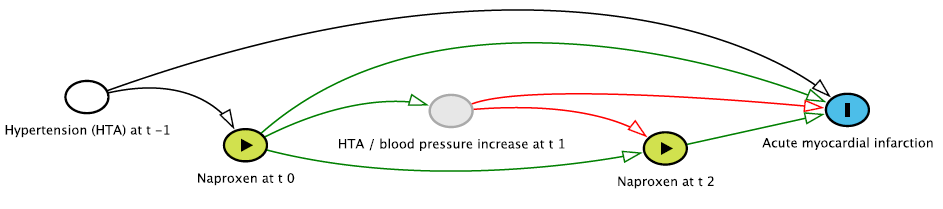


1. Time-dependent confounding by blood pressure affected by previous exposure to the NSAID naproxen. Drawn via the web-based tool DAGitty^[[1]](#footnote-1)^
   NSAID exposures are in green; acute myocardial infarction outcome is in blue; hypertension is a potentially interacting CV risk factor and a confounder of the exposure-outcome association measured at t -1 (confounding path in black); however blood pressure (hypertension) at t1 is unobserved; causal paths are indicated in green; biasing paths are in red

(1)


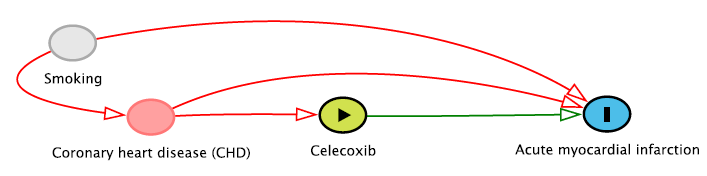

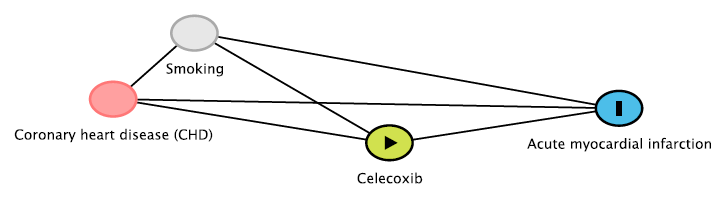


(2)

1. Confounding of the association of CHD and acute MI by smoking
   (1) NSAID exposure is in green; acute myocardial infarction outcome is in blue; CHD is a potentially interacting CV risk factor and a confounder of the exposure-outcome association; smoking is a confounder of the CHD-acute MI association however smoking is unobserved; causal paths are indicated in green; biasing paths are in red
   (2) Same causal diagram as in (1) illustrating correlation paths

1. Textor J, Hardt J, Knuppel S. DAGitty: a graphical tool for analyzing causal diagrams. Epidemiology. 2011;22(5):745.
   DAGitty identifies causal and biasing paths and highlights them in different colors. A color legend helps understand the role of each variable in a causal diagram. [↑](#footnote-ref-1)
